# Evaluation of the Design and Implementation of a Peer-To-Peer COVID-19 Contact Tracing Mobile App (COCOA) in Japan

**DOI:** 10.2196/22098

**Published:** 2020-12-01

**Authors:** Ichiro Nakamoto, Ming Jiang, Jilin Zhang, Weiqing Zhuang, Yan Guo, Ming-Hui Jin, Yi Huang, Kuotai Tang

**Affiliations:** 1 School of Internet Economics and Business Fujian University of Technology Fuzhou China

**Keywords:** COVID-19, contact tracing, mobile app, peer-to-peer, Bluetooth-based, telehealth, privacy protection, load balancing, close contact, decentralized

## Abstract

We evaluate a Bluetooth-based mobile contact-confirming app, COVID-19 Contact-Confirming Application (COCOA), which is being used in Japan to contain the spread of COVID-19, the disease caused by the novel virus termed SARS-COV-2. The app prioritizes the protection of users’ privacy from a variety of parties (eg, other users, potential attackers, and public authorities), enhances the capacity to balance the current load of excessive pressure on health care systems (eg, local triage of exposure risk and reduction of in-person hospital visits), increases the speed of responses to the pandemic (eg, automated recording of close contact based on proximity), and reduces operation errors and population mobility. The peer-to-peer framework of COCOA is intended to provide the public with dynamic and credible updates on the COVID-19 pandemic without sacrificing the privacy of their information. However, cautions must be exercised to address critical concerns, such as the rate of participation and delays in data sharing. The results of a simulation imply that the participation rate in Japan needs to be close 90% to effectively control the spread of COVID-19.

## Introduction

As of August 23, 2020, over 23 million cumulative cases of COVID-19 and nearly 800,000 deaths from the disease have been reported worldwide [1]. Since the first cases were reported in late 2019, the world has witnessed the rapid spread of the pathogen, and it has been declared a global public health crisis by the World Health Organization [2]. Due to the infectiousness of the disease and the dynamics of interperson interactions, the spread of COVID-19 could advance in a way that is unnoticeable to individuals, as evidenced by subclinical presymptomatic and asymptomatic cases [3], which refer respectively to cases in which infection started before the onset of symptoms and infections without the emergence of symptoms. Research has demonstrated the risk of person-to-person transmission of COVID-19 between individuals, especially for those in close contact (ie, close proximity) [4]. When infections are established where individuals are unable to readily self-triage their exposure risk, timely responses to COVID-19 will be challenging; these responses could also be weakened by delayed data sharing, impeded privacy preservation, and impaired security [3].

A variety of measures based on digital health have been adopted to control the spread of COVID-19 [5-13]. These containment measures display heterogeneities in terms of their design: decentralized (ie, mostly privacy-first) versus centralized (ie, mainly data-first) deployment frameworks have emerged alongside Bluetooth-, GPS-, and quick response (QR)–based sensor technologies [5,9]. Countermeasures such as contact tracing can play remarkable roles in the containment of the pandemic, including inference of exposure risk, identification of infections, and quarantining or isolation of individuals being traced [5,10,11]. However, there is major divergence among nations regarding which digital health approach to employ (eg, a centralized approach that collects private data at the expense of potential illegal use vs a decentralized approach that stores data on local devices and leaves individuals in charge of their sensitive information at the cost of constrained accessibility for others) to address critical concerns such as privacy preservation, health care pressure load-balancing, speed of response, and ease of operations [9,12,13]. The centralized approach highlights a data-first methodology and involves the collection of privacy-sensitive information; this approach can enhance the capacity of unified administration, but the identities of individuals can be readily inferred [6,8,9]. GPS- and QR-based contact tracing apps facilitate evidence-based inference and improve the traceability of contact tracing [6,8,9,13]. However, multiple crucial concerns must be addressed to achieve effective containment. The first concern is that the information gathered through GPS is not strictly equivalent to close contact; hence, bias could be undoubtedly introduced. The second concern is the unlawful use or abuse of sensitive personal information obtained using GPS or QR [5-9]. Third, there is some debate that centralized approaches could cause discrimination, reduce confidence, and negatively impact the health of individuals if their private data are misused or breached [9,13]. In contrast, the typical application of the decentralized approach includes Bluetooth-based digital health, which does not theoretically identify individuals; hence, this approach is desirable for settings where concealing users’ identities and preventing accessibility of their contact information are valued by the population [5,8,10,13]. Bluetooth digital health approaches rely on microwave and millimeter-wave technologies to sense the proximity between local devices, enabling the tracking of social contacts with a high degree of precision [5,10,13]. Hence, infections due to close contact (ie, geographical proximity) with pre-asymptomatic or asymptomatic patients can be easily detected and recorded by a Bluetooth-based approach. It is feasible for exposed people to evaluate and self-identify their exposure risk without disclosing either their own identity or the identities of their counterparts. This approach is more rapid and efficient in close contact diagnosis, less labor-intensive, and less susceptible to human error than extant approaches. Bluetooth contact tracing is currently being adopted by numerous countries, including Japan, India, and Singapore [5,9,13-15].

The first cases of COVID-19 in Japan were reported in late January 2020; since then, numerous cases, including asymptomatic and presymptomatic infections, have been identified, and the national medical system has been overburdened, with ever-increasing risk of collapse [14,15]. The excessive strain on the capacity of the medical system is expected to be alleviated. Unidentifiable discrete spreading events could lead to a later outbreak of infections; thus, it is important for individuals to gain an updated understanding of the pathogen [16]. When people can locally track their exposure risk, self-triage, and make differentiated responses based on digitally provided instructions, cross-transmission (eg, cluster infections at crowded locations) and unnecessary in-person visits can be reduced [15].

In this viewpoint, we discuss a decentralized and GPS-free Bluetooth digital health approach, COVID-19 Contact-Confirming Application (COCOA). This approach is mainly used to address the issues of privacy protection, efficacy enhancement, load balancing of pressure on the health care system, population mobility, and manual operation errors, and it principally complies with the Apple and Google contact tracing technology frameworks [5,13]. The major aim is to appraise how the approach can be used as a routine tool to contain the spread of COVID-19, with emphasis on privacy preservation and load-balancing. Prior research has revealed that other factors, such as the rate of participation, play remarkable roles in contributing to the effectiveness of containment [17]. The results of the simulation in our study are consistent with the findings in other empirical research.

## Framework and Core Mechanism of COCOA

### The Architecture and Prototype of COCOA

**Figure 1:**
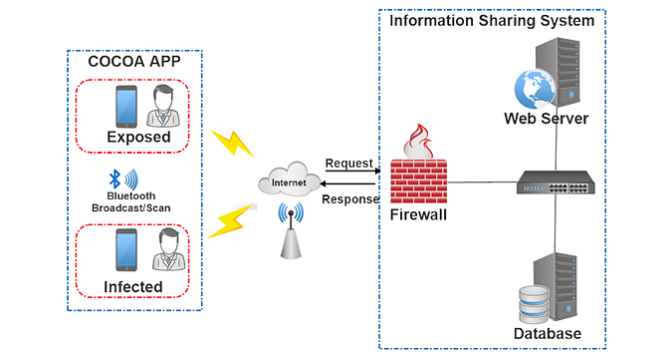
System architecture and prototype of the COCOA app. COCOA: COVID-19 Contact-Confirming Application.

A schematic of the general architecture of the COCOA system is shown in Figure 1. The app automatically records close contact (ie, defined as within 1 meter of proximity for at least 15 minutes in COCOA) on Android and iOS devices by employing Bluetooth technology [18]. The COCOA system consists of three major sections: two mobile terminal apps for individuals (ie, infected and potentially exposed), and an infection information sharing system maintained by public authorities and health care providers. COCOA complies with the decentralized framework; this means that the COCOA app only locally tracks close contacts and performs matching inference of exposure risk, during which no personal private information is requested or collected through COCOA.

### Core Mechanisms

**Figure 2:**
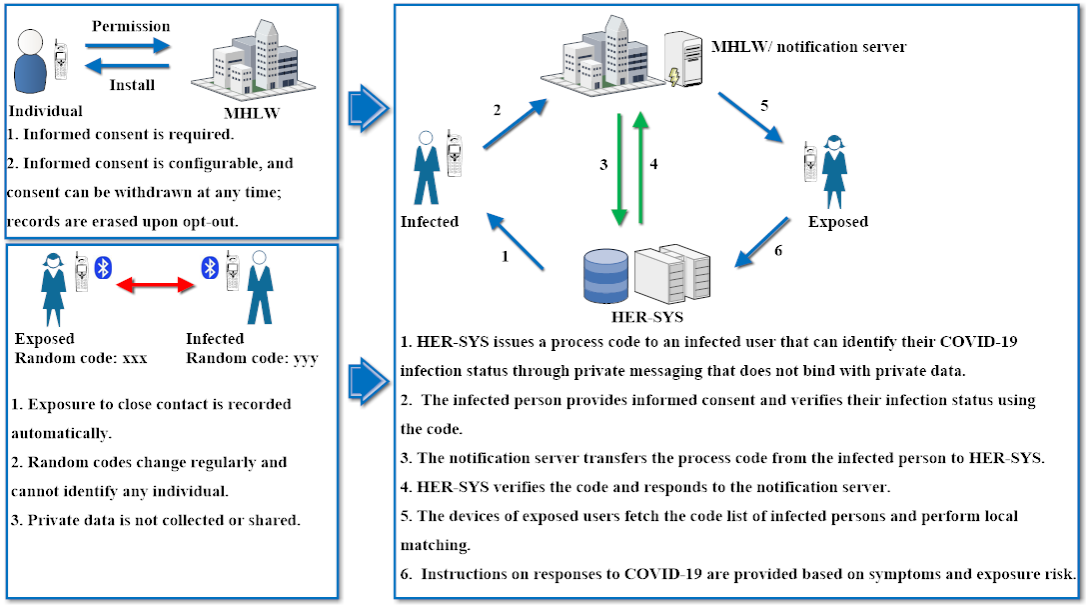
Core mechanisms and diagram of the COVID-19 Contact-Confirming Application (COCOA) framework. HER-SYS: Health Center Real-time Information-sharing System on COVID-19; MHLW: Ministry of Heath, Labor and Welfare.

To conceptualize how COCOA optimizes its core functionality, including privacy protection of individuals and load-balancing of health care stress, in comparison with other centralized digital health approaches, we illustrate the core mechanism and diagram in Figure 2. COCOA integrates the following principal features:

1. Individuals (ie, either infected or potentially exposed) receive informed consent to participate and authorize data sharing.
2. The informed consent feature is configurable, and consent can be withdrawn at any time.
3. Prior records are erased when the user opts out.
4. No sensitive personal information that enables the app user to be identified, such as date of birth, gender, address, telephone number, email address, or location, is requested or collected through COCOA.
5. Close-contact data are encrypted, saved only on users’ local devices, and automatically deleted after 14 days, which is the period generally considered to be the average incubation interval of COVID-19.
6. If the user is infected, informed consent of the COVID-19–positive patient is required to authenticate and distribute their infection status.
7. Upon completion of verification, the process code that is used by the infected person to verify the accuracy of their infection status with the central server is eliminated from the COCOA app, the notification server, and the management system.
8. Exposure risk matching is performed on local devices.

When individuals (whether symptomatic, presymptomatic, or asymptomatic) are in close contact, the COCOA app records this status by automatically exchanging generated random codes, which change periodically and thus cannot be exploited to identify either the infected or potentially exposed users. The codes are not shared with the information sharing system unless the individuals are COVID-19–positive. The codes will be saved only on local devices and erased after 14 days. As these codes are generated randomly and changed periodically, they cannot be exploited to uniquely identify any individuals; this guarantees the preservation of privacy of users’ data from infected people, potentially exposed people, attackers, and public authorities. In this way, concerns regarding privacy-preserving issues inherent to other technologies (eg, GPS and QR) can be waved. The detection of close contacts can run automatically in the background without requiring COCOA to be active, all of which can be unnoticeable to the individuals in contact. This feature improves the efficacy of detection, increases the ease of operations, and reduces manual errors [15,18].

The Health Center Real-time Information-sharing System on COVID-19 (HER-SYS) is operated and maintained by prefecture-level or local health care providers. It issues a process code when an individual tests positive for COVID-19 by polymerase chain reaction (PCR). These process codes are distributed to the patients through private messaging (eg, emails) and are not exploited to bind private information (eg, telephone numbers that can identify individuals). Hence, privacy protection issues during the data dissemination steps are also not of concern. The notification server is administered by the Ministry of Health, Labor and Welfare in Japan, and its functionality is considerably constrained for privacy protection. The notification server does not store the patients’ infection status or any other sensitive personal information. When one individual is notified that they have been infected, they are encouraged to share their status with potential close contacts. However, informed consent and authorization are requested. To prevent malicious inquiries and to guarantee the accuracy of data, the patient must input a process code and authenticate the correctness of their data with the notification server, which transfers the request to HER-SYS. HER-SYS authenticates the accuracy and returns the outcome to the notification server, which then distributes the random code of the infected person to all potentially exposed people upon request. The COCOA apps of the exposed people then perform local matching inference based on the retrieved anonymized list of random codes for individuals infected with COVID-19. Note that asymptomatic or presymptomatic infections can be traced effectively because the detection mechanism of COCOA hinges on geographical proximity, which is generally considered to be the critical factor contributing to the transmission of highly infectious disease. If a match is found and the exposure risk is identified, health care instructions on the outcome and the severity of risk will be provided. The potential exposed person can respond appropriately according to their corresponding symptoms. For severely symptomatic individuals, urgent care must be scheduled. In contrast, asymptomatic or mild cases may choose to self-isolate at local sites. As the matching calculation is performed locally and individuals can self-triage the exposure risk, risky in-person visits and cross-transmission at health care facilities can be curtailed. This can prioritize limited health care resources for more severely ill patients and enhance the load-balancing of the pressure on medical systems, thus lowering the risk of collapse of the health care system [14,15,18].

Different countries differ in their digital health participation rates [9,12,13]. According to a report by MIT Technology Review [19], we outlined the statistics of countries adopting or partially adopting Bluetooth digital health by the end of July 2020 (Table 1). On average, the rate of participation is higher for countries using centralized Bluetooth digital health frameworks than for countries using decentralized Bluetooth frameworks; however, the latter frameworks generally outperform the former in privacy protection. Further, countries using decentralized approaches, most of which hinge on voluntary participation, mostly sustain low rates of participation. None of these countries currently have a participation rate ≥60% [19].

**Table 1:** Estimated participation rates of countries employing Bluetooth frameworks.

Country	Participation rate (%)	Centralized or decentralized
Japan	12.6^a^	Decentralized
Australia	25.8	Centralized
Austria	6.8	Decentralized
Bahrain	25.5	Centralized
Czech	2.6	Decentralized
Fiji	3.1	Decentralized
France	2.8	Centralized
Germany	16.9	Decentralized
Gibraltar	26.7	Decentralized
Hungary	0.10	Centralized
India	7.39	Centralized
Indonesia	7.10	Centralized
Italy	3.64	Decentralized
Malaysia	0.32	Decentralized
New Zealand	12.1	Centralized
Norway	26.6	Centralized
Philippines	1.1	Decentralized
Poland	0.1	Decentralized
Qatar	91	Centralized
Singapore	37.2	Centralized
Switzerland	5.8	Decentralized
Thailand	5.1	Decentralized
Tunisia	0.2	Centralized
Turkey	17.3	Centralized
Vietnam	0.4	Decentralized

^a^Data for Japan as of August 27, 2020.

To illustrate how countries using Bluetooth digital health can differ in the capacity of privacy preservation, we provide details of two centralized frameworks: one voluntary (ie, Aarogya Setu in India [20]) and one involuntary (ie, TraceTogether in Singapore [21]). Emphasis is placed on the core qualitative concepts employed to clarify the major differences; detailed quantitative technical specifications are not examined in this paper (Textbox 1).

**Core concepts of the COVID-19 Contact-Confirming Application (COCOA) framework and of Bluetooth digital health frameworks in other countries.**

COCOA (Bluetooth-based digital health framework in Japan)

- Personal information, including names, telephone numbers, and GPS locations, is not requested or collected.
- Participation is voluntary. Informed consent to participate is requested.
- Close contact detection automatically runs in the background without requiring COCOA to be active, resulting in ease of use and low power consumption.
- Mobile phones generate and exchange periodically changing random codes with close contacts.
- Close contact information is saved only on local mobile phones for 14 days and is not transmitted. Individuals poll the central server (without sharing private information) to retrieve the list of infected people, not the reverse.

Aarogya Setu (Bluetooth-based digital health framework in India)

- Personal data, including name, gender, travel history, and telephone numbers, are requested and shared with the central server.
- GPS locations are collected and used to trace the paths of infected individuals.
- Participation is voluntary.
- There are risks of data inaccuracy and illegal data use.
- It is difficult to operate.
- Its power consumption is high.

TraceTogether (Bluetooth-based digital health framework in Singapore)

- Random tokens recording close contacts are shared with the central server, which maintains a database linking tokens and telephone numbers. There is a likelihood of linkage attacks and unlawful use.
- Infected individuals are required by law to share their infection status, including telephone and unique identification numbers.
- Individuals are notified of their exposure risk via identifiable information (eg, telephone numbers).
- GPS location data are not tracked; however, telephone and unique identification numbers are collected by public authorities.
- Participation is generally mandatory.
- Inference of exposure match is performed on the authority-administered central server.

COCOA differentiates from Aarogya Setu in privacy preservation in that the latter framework requests individuals’ self-reported personal data, including gender, travel history, and telephone numbers; this raises concerns regarding the accuracy of data in the case where users share wrong information, as no mechanism is provided for authentication. Further, because GPS locations are collected by Aarogya Setu, it is debated that these data could be used to identify individuals without improvement of contact tracing precision (eg, individuals on different floors of the same building) [20]. In contrast, in Singapore, individuals are legally required to share their infection status; hence, the rate of participation can be guaranteed. However, personal data such as telephone numbers and unique identification numbers are also collected, which creates concerns regarding the possibility of illegal use of private information. Further, inference of exposure is performed on the central server; hence, technical pressure on the medical system must be optimized [21].

### Screenshots and Diagram of the COCOA App

**Figure 3:**
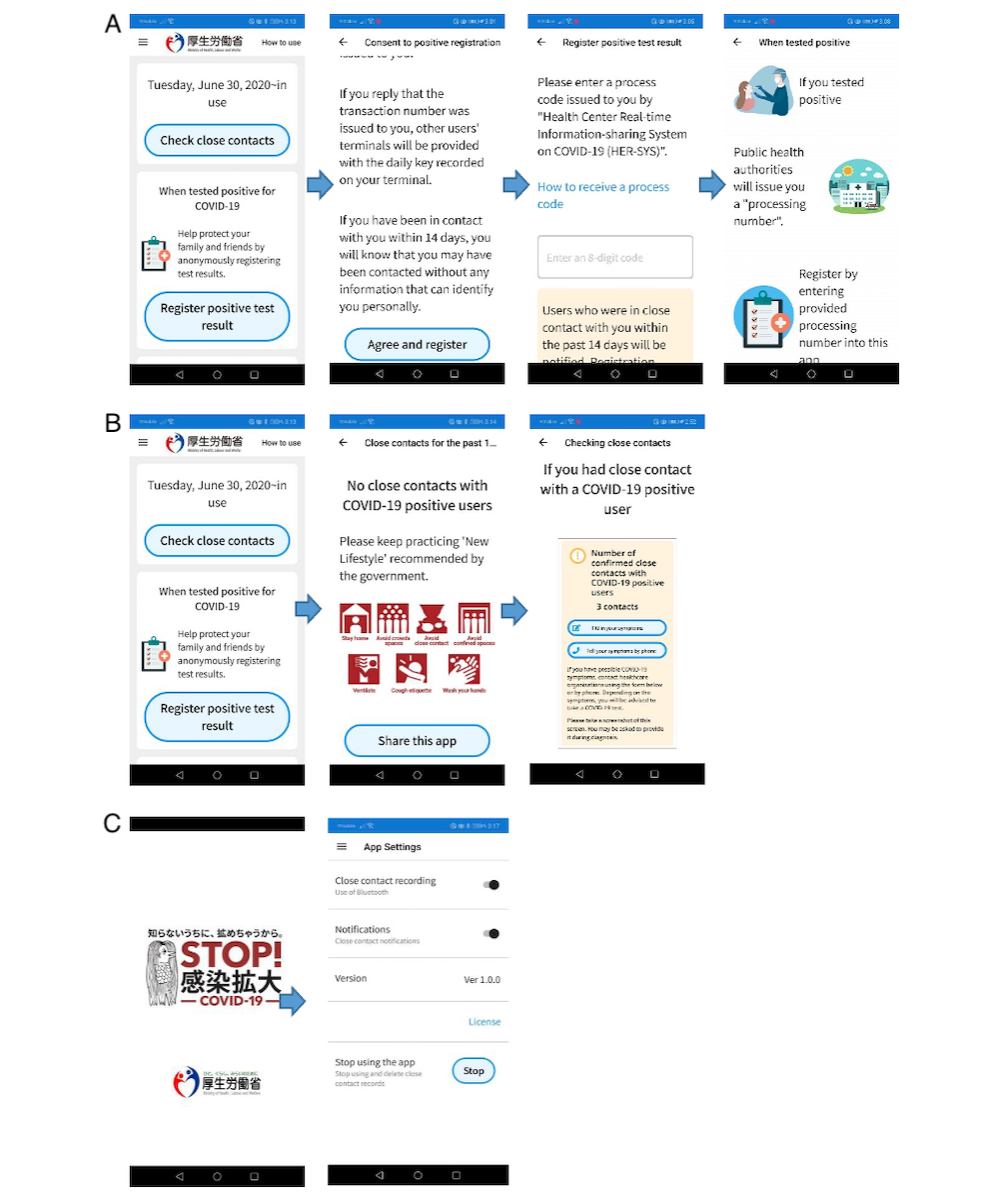
Screenshots and diagram of the use of the COVID-19 Contact-Confirming Application (COCOA) app. (A) Infected users verify their infection status and authorize data sharing; (B) potentially exposed users retrieve the infection list and triage their exposure risk; (C) users can adjust their close contact settings.

Figure 3 shows screenshots of the COCOA app. The app can be divided into components for infected people (Figure 3A), potentially exposed people (Figure 3B), and general settings (Figure 3C). The process can be described as follows:

1. An individual is tested and identified as COVID-19–positive using PCR. The individual receives a process code from HER-SYS, verifies the accuracy of their status through the notification server, and authorizes anonymized sharing of infection status (Figure 3A).
2. Potentially exposed individuals retrieve the up-to-date list of COVID-19 infections (Figure 3B).
3. If an exposure match is identified, exposure statistics and subsequent response guidance are promptly provided. Otherwise, a message indicating no exposure is promptly provided (Figure 3B).
4. Close-contact recording and COCOA participation are configurable. Records will be erased if the user opts out (Figure 3C).

## Statistical Analysis and Simulation Results

**Figure 4:**
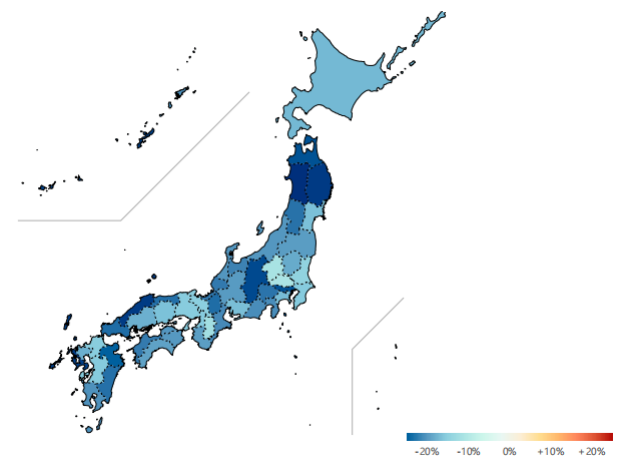
Comparison of the population mobility in Japan in August 2020 versus August 2019.

Digital health can enhance individuals’ knowledge and risk perceptions of COVID-19, thus decreasing population mobility [22-25]. Reducing mobility is a controversial but effective measure to flatten the curve and control global pandemics; it decreases the generation of crowded spaces that aggravate cluster infections [23-25]. A simulation was conducted that implies that the spread of COVID-19 in Japan will be gradually contained by reducing the population mobility and the amount of time spent in crowded spaces [22]. We observed the dynamics of population mobility at the prefecture level in Japan by comparing data from August 2020 (ie, when COCOA was deployed) with data from the same interval of the previous year (ie, when COCOA was not deployed) (Figure 4). The analysis suggests that the nationwide population mobility in 2020 decreased by 20% on average (ranging from the minimum of 12% in Saitama Prefecture to the maximum of 30% in Akita Prefecture). Reducing population mobility lowers the risk of exposure to COVID-19 and the risk of infection [18,25,26].

Since its official deployment on June 30, 2020, COCOA has been used by approximately 15 million individuals as of August 27, 2020 [18]. This finding denotes that around 12.6% of the population of Japan (15 million/118.6 million people aged ≥15 years) chose to participate by August 2020 [27]. The intervention measures deployed in Japan are noncompulsory, informed consent is requested prior to participation, and no legal penalty is imposed in the case of noncompliance; these features are less aggressive than those of digital strategies in some other countries [22-25].

In Japan, 69,001 confirmed cases of COVID-19 and 1307 deaths were reported from January 14 to September 2, 2020; the numbers of cases and deaths during this time were 3,769,523 and 66,333 for India and 56,852 and 27 for Singapore, respectively [26]. The effectiveness of containment in Japan is greater than that in India in terms of both infected cases and mortality. However, countries with compulsory contact tracing measures (eg, Singapore) appear to outperform those with noncompulsory measures (eg, India and Japan). It was estimated that older people (ie, age ≥65 years) in Japan would comprise 28.7% of the total population by the end of August 2020 [18,25-27]; the rates of severe cases and mortality among older people were 2.0% and 1.9%, respectively, during the same period, which are lower than the global average mortality rate of 3.3% (852,758/25,602,665) [18,26].

**Figure 5:**
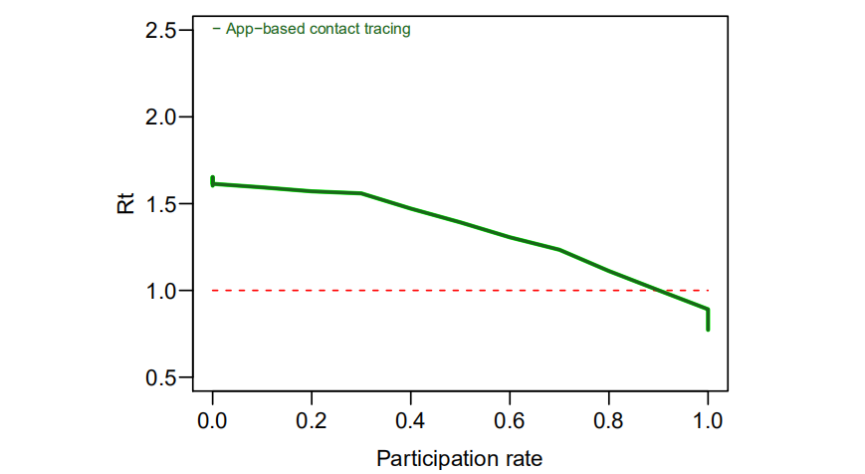
Simulation of the association between the rate of participation in the COVID-19 Contact-Confirming Application (COCOA) framework and the R_t_ of COVID-19. R_t_: effective reproduction number.

A simulation performed at Oxford University suggested that digital contact tracing would fail to decrease the spread of COVID-19 if the rate of participation fell to <60% (600,000/1,000,000) [28]. Similar analyses by other researchers reinforce that varying adoption rates of peer-to-peer contact tracing apps can influence the trajectory of the pandemic [17,29]. By employing the simulation model described in [30], we estimated how the rate of participation would affect the trajectory of the COVID-19 pandemic in Japan (Figure 5). The model evaluates scenarios in which the epidemic is established and countermeasures such as contact tracing are employed to control the spread of COVID-19; it can be observed how the trend of the effective reproduction number (R_t_) and thus of the outbreak would dynamically change. Prior research identified that when R_t_, which is defined as the average number of secondary cases generated by a single infectious case, decreases to less than one (ie, R_t_<1), transmission of the disease will stop and the pandemic will ultimately be contained [22-24]. The simulation was calibrated to the demographic attributes in Japan [14,22,25-27,29], the basic reproduction number (ie, 2.56) found for Japan [30], and the ratios of symptomatic patients in the report by the National Institute of Infectious Diseases in Japan [31]. The assumptions for the simulation are as follows: (1) the population can freely choose to opt in or opt out from COCOA; (2) there are no delays in data sharing; and (3) all the populations in households, schools, workplaces, and other scenarios can be successfully digitally traced. The simulation outcome (Figure 5) shows that when the participation rate increases starting from zero, the effective reproduction number decreases gradually from a value >1. However, the pandemic would finally be contained when a threshold was exceeded and more people chose to opt in. To meaningfully contain the spread of COVID-19 (ie, R_t_<1), approximately 90% participation of the population would be required, which reinforces prior findings that controlling COVID-19 requires an estimated population uptake ranging from 56%-95% for contact-tracing apps [32].

## Strengths, Limitations, and Future Directions

The COVID-19 pandemic has imposed unprecedented challenges upon individuals, health care providers, and public authorities; meanwhile, different countries are using differentiated digital technologies and strategies to contain the spread of COVID-19, taking into account both technical and nontechnical factors. Digital health is not a panacea that will solve all difficulties; however, it does enhance the potential to counteract the disease compared to manual contact tracing [33,34].

In comparison with the Bluetooth-based digital health frameworks in other countries (eg, India or Singapore) or centralized approaches, COCOA more effectively protects the privacy of individuals from their counterparts, potential attackers, and public authorities without sacrificing accuracy or efficiency. First, users are not tapped to self-report personal data (eg, names or telephone numbers) through the app; this enhances the efficacy and eliminates the need for proofreading when users input incorrect data. Additionally, concerns about malicious use or illegal breach of private data can be waived. Moreover, persons are not requested to share their private information when infected or provide sensitive data, which may be subject to linkage attacks, when notified of infection. The authentication of infection precludes malicious exploitation of or attack on accurate infection information by other individuals, preventing misinformation regarding exposure. Location details, which are an unsuitable proxy for exposure, are not collected; this ensures that the movement paths of individuals will not be tracked and the identities of the individuals will hence not be disclosed.

Further, because the matching of exposure inference is performed on local sites, and subsequent provision of instructions when exposure risk is identified can curtail unnecessary in-person visits and risk of crosstransmission, the load of pressure on the health care system can be substantially balanced [9]. Bluetooth digital health uses proximity to identify close contact; hence, the speed of close contact detection is faster than that of other non-Bluetooth digital approaches. COCOA can run automatically in the background without interfering with other apps, which reduces errors from manual operation and enhances its efficiency. The deployment of the app contributes to increased risk perception and the reduction of nationwide population mobility.

Contact tracing is an essential part of transitioning back to normal economic rhythms while simultaneously managing the risk of subsequent cyclical outbreaks [9,33-35]. The benefits can be multiple for a variety of responders. For potentially exposed or infected individuals, it is possible to know whether risky exposure has been established or disseminate knowledge to others without disclosure of identity or leakage of confidential information, which can increase individuals’ confidence and trust in the health care system and self-awareness of their own behavior changes. This may facilitate appropriate and timely responses to the disease. For potential attackers, as no private information is available regarding either infected or exposed people for illegal or unauthorized exploitation, abnormal activities inherent to centralized or other digital health frameworks can be waved. For public authorities, the triage of patients and cumbersome matching inference of exposure are significantly trimmed; hence, the pressure on the medical system is expected to be alleviated. Further, as public authorities do not store personal private information, the risk from any attack on or misuse of their data is minimized. Bluetooth digital health has great potential to be used as a routine and mainstream tool in future outbreaks [36]. The rate of participation is expected to increase over time. COCOA supplies a new approach that is supplemental to extant digital health frameworks that fail, either partially or completely, in these facets. It could perform or match well in contexts where the population is highly privacy-sensitive and where limited health care resources are at risk of collapse.

Although decentralized telehealth has a variety of benefits and strengths, it has disadvantages as well [33,34,37]. Multiple critical concerns must be addressed to achieve effective containment.

First, the participation rate can essentially affect the trajectory of the outbreak. Studies have shown that societal level benefit hinges on broad and diverse user participation [19,38-43]. A low rate of participation can be associated with factors such as users’ altruism, the population in rural or remote areas, wireless connectivity, availability of digital health, level of digital illiteracy, and legitimation regulations. In some countries, the use of private data is protected legally; whether this applies to other settings may need require more study and more time [5,9]. Solutions that have performed well for some communities may not work well in other communities with different cultural norms, legitimate regulations, and shared perceptions of privacy. From the legislation perceptive, public disclosure of individuals’ protected data may be a violation of law in some contexts [39]. A higher level of participation may be achieved through mandatory legal regulations, enforcing adoption or substantial enhancement of shared public awareness. However, the rapid adoption of compulsory digital health measures without public consensus and discussions could provoke debates due to the fundamental heterogeneity in the attitudes regarding how digital health should function and, crucially, who should have access to the generated data [38-42,44,45]. Residents’ perception of privacy and trust in public authorities can vary from culture to culture, which can impact the captured definition of individual privacy preservation [39-41]. A survey conducted in five other countries (ie, France, Germany, Italy, the United Kingdom, and the United States) found that people in settings with stronger public privacy and security concerns are relatively less supportive of app-based contact tracing, and individuals with less trust in public authorities are also less supportive [46,47].

Second, the delays in data sharing could allow the spread of COVID-19 to continue, increasing the time and effort needed to contain it [9,46,47]. If infectious individuals and their close contacts could be identiﬁed with efficacy, the effectiveness of digital health could be increased remarkably, and limited health care resources could thus be prioritized for the quarantining and treatment of the most severe cases [42]. However, this mechanism is compromised during a pandemic, in which delays of data sharing occur. Voluntary participation could cause noncompliance, generating a latency in responses [42,43]. The spread of COVID-19 hinges partially on the efficacy of data sharing and promptness of responses, given the infectiousness of the pathogen [42]. The greater the delays, the more difficult it is to contain the outbreak. Hence, timely sharing of information is critical to prevent subsequent cyclical outbreaks [43]. Finally, as data are automatically erased after a periodic interval, it is difficult to evaluate the long-term effects of a decentralized Bluetooth approach [13].

Future research could examine how privacy-enabled noncompulsory Bluetooth digital health can both quantitatively and qualitatively reduce the effectiveness of contact tracing relative to compulsory interventions. It could also examine ways to improve critical factors such as participation rate and delays of data sharing in these settings to enhance the effectiveness of containment. With the combined efforts of a variety of responders, the negative impacts of these factors are expected to be minimized. Coupled with the advancement in digital technologies and scientific understanding, telehealth can be enhanced to serve as a sustainable and mainstream solution to counter the COVID-19 pandemic, and it can be simultaneously employed as a routine tool to protect the privacy and well-being of the public [16].

## Conclusions

The balance between privacy protection, public health, and other objectives is controversial [13]. COCOA contributes to prioritizing the preservation of users’ privacy more effectively than the centralized Bluetooth digital health frameworks used in some other countries. The matching inference of exposure is performed locally, and individuals can self-triage their risk of exposure, which facilitates the load balancing of pressure on the medical system. It works better in load-balancing than centralized frameworks. As public authorities do not collect or manage users’ sensitive personal information, concerns regarding illegal use or malicious attacks on private data can be disregarded. The detection of close contact is rapid and effective, and it reduces the likelihood of crosstransmission and in-person contacts. The background running feature enhances the efficacy of the approach and reduces errors of operation, which could be vital in the fight against highly infectious diseases such as COVID-19.

Since the deployment of COCOA, an average of 20% reduction in population mobility has been observed in Japan, which has affected the trajectory of the outbreak. With the wide spread of wireless connections and advancements in digital technologies, digital health can reduce inequality in access to health resources, promote health literacy, and improve risk perceptions. The Tokyo area has observed faster growth in the number of infected cases than other prefectures in Japan [22,24]; hence, substantial improvements in the participation rate and speed of data sharing are of great concern in these densely populated communities or in places where the risk of close contact is high [22-24].

Countries diverge in their digital health frameworks and technologies. Decentralized privacy-first Bluetooth approaches can protect citizens’ sensitive information, but possibly at the expense of compromised participation and impeded central surveillance. In contrast, a centralized data-first framework can warrant traceable data but may substantially violate individuals’ privacy. Cultures differ in the perception and definition of privacy. The lack of a consensus on privacy protection in contact tracing incurs risks of noncompliance, as evidenced by recent privacy scandals [42,43]. This has hindered governments’ capacity to effectively respond to the pandemic. The deployment and acceptance of telehealth in specific settings reflect both technical and nontechnical factors such as regional heterogeneity, cultural conflicts, shared altruism, and legal regulations [9,44].

Given that participation and data sharing are nonbinding, the privacy-first approach could consistently generate skepticism but ideally will enable the implementation to mitigate current and subsequent cyclical pandemics [41]. Coupled with the efforts from a variety of responders, the rate of participation and delays in data sharing are expected to improve over time. Countries using the decentralized Bluetooth approach must prioritize deliberation of how currently unresolved problems can be addressed to contain the spread of COVID-19. Digital health itself cannot overcome all these challenges; however, by combining it with other countermeasures, such as social distancing, early case isolation, and hygiene practice, it is feasible to achieve meaningful containment [45]. With these improvements, it could be feasible to achieve a balance between privacy preservation and public health by enabling individuals to have full control over sensitive data, identify local exposure risk, share their data in a timely fashion, and enact prompt responses [42].

This decentralized Bluetooth approach will undoubtedly upgrade its definition with advancement in digital health, digital technologies, and a more accurate scientific understanding of the disease. Lessons learned from this current deployment will play paramount roles in future pandemics, further aid the establishment of an effective routine surveillance approach, and provide meaningful insights for other countries and regions.

## Abbreviations

COCOA: COVID-19 Contact-Confirming Application
HER-SYS: Health Center Real-time Information-sharing System
PCR: polymerase chain reaction
QR: quick response
R_t_: effective reproduction number
